# Genetic Characterization of Russian Rapeseed Collection and Association Mapping of Novel Loci Affecting Glucosinolate Content

**DOI:** 10.3390/genes11080926

**Published:** 2020-08-12

**Authors:** Rim Gubaev, Lyudmila Gorlova, Stepan Boldyrev, Svetlana Goryunova, Denis Goruynov, Pavel Mazin, Alina Chernova, Elena Martynova, Yakov Demurin, Philipp Khaitovich

**Affiliations:** 1 Skolkovo Institute of Science and Technology, Moscow 121205, Russia; rim.gubaev@skoltech.ru (R.G.); S.Boldyrev@skoltech.ru (S.B.); S.Goryunova@skoltech.ru (S.G.); D.Goryunov@skoltech.ru (D.G.); P.Mazin@skoltech.ru (P.M.); alin.chernova@gmail.com (A.C.); E.Martynova@skoltech.ru (E.M.); 2 Pustovoit All-Russia Research Institute of Oil Crops, Krasnodar 350038, Russia; lagorlova26@yandex.ru (L.G.); yakdemurin@yandex.ru (Y.D.); 3 Institute of General Genetics, Russian Academy of Science, Moscow 119333, Russia; 4 FSBSI Lorch Potato Research Institute, Kraskovo 140051, Russia; 5 Belozersky Institute of Physico-Chemical Biology, Lomonosov Moscow State University, Moscow 119992, Russia

**Keywords:** rapeseed, genotyping-by-sequencing, genetic diversity, population structure, glucosinolates, association mapping

## Abstract

Rapeseed is the second most common oilseed crop worldwide. While the start of rapeseed breeding in Russia dates back to the middle of the 20th century, its widespread cultivation began only recently. In contrast to the world’s rapeseed genetic variation, the genetic composition of Russian rapeseed lines remained unexplored. We have addressed this question by performing genome-wide genotyping of 90 advanced rapeseed accessions provided by the All-Russian Research Institute of Oil Crops (VNIIMK). Genome-wide genetic analysis demonstrated a clear difference between Russian rapeseed varieties and the rapeseed varieties from the rest of the world, including the European ones, indicating that rapeseed breeding in Russia proceeded in its own independent direction. Hence, genetic determinants of agronomical traits might also be different in Russian rapeseed lines. To assess it, we collected the glucosinolate content data for the same 90 genotyped accessions obtained during three years and performed an association mapping of this trait. We indeed found that the loci significantly associated with glucosinolate content variation in the Russian rapeseed collection differ from those previously reported for the non-Russian rapeseed lines.

## 1. Introduction

Rapid development of high-throughput genotyping technologies, as well as the availability of assembled plant genomes, substantially facilitated the analysis of genetic variation and search for loci associated with agronomically important traits in crop species [1]. Rapeseed (*Brassica napus*) is the second crop most widely cultivated with the purpose of vegetable oil production [2]. Rapeseed genome is sequenced and annotated [3,4], which makes it a perfect model organism to study genetic diversity and to reveal the genetic bases of complex phenotype traits on a genome-wide scale with high resolution. Thus, a number of studies have been performed in order to find genetic associations for the agronomically important traits related to oil quality, plant productivity and architecture, and resistance to unfavorable environmental conditions by means of the modern genotyping approaches in rapeseed [5,6,7]. In addition, several studies were focused on the description of genetic diversity, evolution, domestication footprints, and population structure of the rapeseed [8,9,10,11,12].

Notably, most of the present day studies focusing on association mapping were conducted using microarray-based genotyping, due to the availability of the high-quality *Brassica* 60k Illumina array that has been developed recently [13]. Nevertheless, sequencing-based approaches have also been utilized in the recent studies [9,10,14]. The use of sequencing-based genotyping approaches allows, in some cases, to reduce genotyping costs and to detect rare or previously unknown genetic variants [15,16,17].

One of the key agronomically important traits related to the quality of rapeseed oil and oilcake used as animal fodder is glucosinolate content [18,19]. Glucosinolates constitute a group of secondary metabolites produced by the *Brassicaceae* family. These metabolites, on the one hand, facilitate plant defense against pathogens and, on the other hand, contribute to the bitter taste of *Brassicaceae* plants [19]. Furthermore, glucosinolates were shown to negatively affect the kidney and liver in higher animals [18]. Hence, rapeseed breeding programs are in general aimed at reducing the presence of glucosinolate components in rapeseed oil and oilcakes [18].

To date, several studies have been performed in order to find genetic associations for glucosinolate content in rapeseed. These studies included the QTL mapping approach using double haploid populations [20,21], as well as the ones using F_2_ mapping populations derived from the lines contrasting in glucosinolates [22,23]. These studies identified a number of genetic loci associated with glucosinolate content located on the chromosomes A01, A04, A06, A09, C02, C03, and C09. Following the increasing popularity of GWAS several works have been published on implementing this method to diverse rapeseed lines from global collections [24,25,26]. These approaches facilitated the identification of new loci as well as new candidate genes potentially responsible for glucosinolate content in rapeseed plants.

Despite the numerous studies focusing on genetic diversity, as well as on high-throughput genotyping-based association mapping of glucosinolate content, most of these studies did not include Russian rapeseed lines. It could be related to the fact that rapeseed production in Russia started gaining momentum only in the beginning of the 21st century [27]. Thus, there are almost no studies devoted to rapeseed performed on a genome-wide scale. Our study focused on the high-throughput genotyping of a significant number of diverse rapeseed accessions from the collection of the All-Russian Research Institute of Oil Crops (VNIIMK), one of the leading breeding institutes and rapeseed varieties producers in Russia [28]. The VNIIMK collection used in the present study included 90 lines, most of which are of Russian origin or were obtained by crossing international lines with Russian ones. Furthermore, the studied cohort also included several lines of European and Chinese origin. Thus, this material represents not only Russian lines but also several international ones currently used by Russian and VNIIMK breeders, in particular, to obtain new valuable varieties used for vegetable oil and animal fodder production. In the present study, we aimed to describe the population structure of VNIIMK collection based on the genotype data as well as to compare it with the accessions from the global rapeseed collections. We also looked for genetic associations with agronomically important traits, namely, glucosinolate content, in order to facilitate rapeseed marker-assisted selection in VNIIMK and in Russia in general.

## 2. Materials and Methods

### 2.1. Plant Material and Phenotyping

The plant material for the present study consisted of 47 spring and 43 winter inbred diverse rapeseed lines from the collection of All-Russian Research Institute of Oil Crops (VNIIMK). The phenotype data were collected at the VNIIMK experimental station in the Krasnodar region, Russia, during three (2015–2016, 2016–2017, 2018–2019) vegetation periods. GPS coordinates were 45°10′51″ N and 39°02′42″ E. Soil type was chernozem alkali soils.

To measure the glucosinolate content, a modified palladium colorimetric analysis [29] was performed. For each measurement, 5 g of seeds were collected from three plants of each line from a single plot during each of the three years. Seeds were homogenized using a mechanical grinder, then 0.2 g of the homogenate were used for extraction by the addition of 0.3 mL of 80% methanol with subsequent incubation for 5 min at 90 °C. After the incubation, 3 mL of boiling water was added with subsequent incubation for 20 min at 90 °C. Then, 0.1 mL of a 30% ZnS0_4_ solution and 0.1 mL of a 15% K_4_[Fe(CN)_6_]·3H_2_O solution were added, with thorough stirring after adding each solution. The obtained solution was diluted to 5 mL with distilled water. The prepared extract was thoroughly mixed and filtered using a qualitative filter paper. The 0.2 mL of the filtered extract was added to 1.8 mL of tetrachloropalladate (II) (PdCl_2_). After incubating at room temperature for 30 min, absorbance at 420 nm was measured using a spectrophotometer. The concentration was calculated based on the calibration curve obtained using the standard samples with known concentrations. The data on the line name, ecotype, origin, and glucosinolate concentrations are summarized in Table S1.

### 2.2. Genotyping Procedures

For genotyping procedures, three independent plants from each line were used. As a result, 270 individual plants were genotyped. For DNA extraction, 1–2 leaves were obtained from the seedlings after two weeks of germination at room conditions. Total DNA was extracted according to the CTAB protocol using the NucleoSpin Plant II plant DNA extraction kit (Macherey-Nagel, Dylan, Germany) and stored at −20 °C until needed. The quality and concentration of the purified DNA samples were determined by gel electrophoresis and in the Qubit 3.0 Fluorometer (Thermo Fisher Scientific, Waltham, MA, USA). The genotyping-by-sequencing (GBS) library was prepared according to the previously described protocol [30] using PstI and MspI restriction enzymes for DNA digestion. GBS library sequencing was performed in the HiSeq 4000 (Illumina, San Diego, CA, USA). Raw sequence data are available on NCBI SRA under the project number PRJNA645178. The line name, sample, and corresponding barcodes are summarized in Table S2.

### 2.3. Read Filtering, Alignment, and SNP Calling

Read demultiplexing was performed using the Axe-demultiplexer software version 0.3.3 [31]. Demultiplexed reads were filtered using the Trimmomatic software version 0.36 [32]. Low-quality bases (Phred score < 20) in the reads, as well as reads that correspond to Illumina adapters and short reads (<40 bp) were removed. Filtered reads derived from the plant samples of the same line were merged and then aligned to the ZS11 rapeseed genome assembly [4] using the Burrows-Wheeler aligner software version 0.7.17 [33] with default settings. For SNP calling, the Genome Analysis Toolkit software version 4.1 [34] was utilized. The biallelic SNPs that passed the following filters were used for subsequent analysis: Minor allele frequency (MAF) > 0.03, maximum heterozygosity of 40%, and maximum missing data of 30%. The sequencing depth was of at least 10 reads per position.

### 2.4. Population Structure and Genetic Diversity Assessment

In order to find the number of subpopulations within the studied cohort of rapeseed lines, ADMIXTURE software version 1.3 [35] was used. Linkage disequilibrium was evaluated using *r^2^* which in turn was derived from the pairwise comparisons made in the PLINK software version 1.9 [36]. The *r^2^* value was calculated for SNP pairs located within 1500 kb frames across A and C subgenomes as well as for the whole genome (A + C subgenomes). Furthermore, the proportion of SNP pairs with the *r^2^* value greater than 0.25 within 30 kb bins was also estimated for the whole genome. For the diversity analysis, the whole-genome sequencing data of 54 rapeseed inbred lines (Table S1) from the global collections [37] were used. Lines selected for diversity comparison were from different geographic regions (including lines from European, Chinese, Australian, Japanese, Moroccan, and Indian collections) and of different ecotypes (winter, semi-winter, and spring). Such contrast set was used for better representation of the world’s diversity of the rapeseed lines for subsequent comparison with the Russian collection that includes several lines of international origin as well as lines of both spring and winter ecotypes. Read filtering, alignment, and SNP calling was performed with the parameters described above. To find SNPs common for the studied cohort and the global collection, the Bcftools software version 1.9 [38] was used. The principal component analysis was performed using default parameters in the PLINK software version 1.9. The number of principal components was set to 10. Principal components were estimated using the variance-standardized genetic relationship matrix.

### 2.5. Association Mapping, SNP Annotation, and Loci Comparison

Association mapping was performed using the TASSEL software version 5.2 [39]. The mixed linear model (MLM) approach with a compression was applied to reveal the genotype-phenotype associations. The first five principal components, as well as the kinship matrix calculated using the centered IBS methods, were added to the model to account for the population structure and kin relationships, respectively. MLM was used to generate the *p*-values for SNPs using the phenotype data for each year independently. To find the most reproducible SNPs across three years, the sum of the *p*-values was substituted by the corresponding densities of the Irwin-Hall distributions taking into account that the sum of *p*-values was formed by three uniform distributions. For this procedure, the Dirwin.hall function from the Unifed R package was used [40]. In order to assume an association, the adjusted *p*-value (Bonferroni correction) of less than 0.0000041 (0.05/12,226) was used, where 12,226 is the number of tests (SNPs). The second softer threshold was set to 0.0005. In order to scan for the genes located close to the SNPs significantly associated with the glucosinolate content, the SnpEff software version 4.3 was utilized [41]; genes located 100 kb upstream and downstream of the SNPs were searched for.

In order to compare the newly identified loci (SNP positions) with the loci that were previously demonstrated to be significantly associated with glucosinolate content, first, the available information on the exact physical distance of loci associated with glucosinolate content was collected from the previously published papers [24,25,26,42,43]. Second, for each SNP in the present study, the corresponding positions in the Darmor bzh assembly version 4.1 [3] were found. This was done because the previous studies were carried out using this assembly. To do this, the flanking sequences of 50 bp upstream and downstream the regions of the SNPs were extracted in order to find the corresponding positions in the Darmor bzh assembly version 4.1 with the aid of the Blast software version 2.2.18 [44]. The following filters were applied in order to find the SNPs common between the two assemblies: Alignment length > 90, *e*-value < 1 × 10^−40^. The frame of 100 kb was used to find the genetic regions in common with the previous studies.

## 3. Results

### 3.1. Population Structure and Genetic Diversity Assessment

We assessed the genetic variability of the Russian rapeseed collection using the genotyping-by-sequencing approach. For each of the 90 rapeseed lines, we genotyped three independent plants using the Illumina paired-end sequencing procedure. Three independent plants were used in order to increase the confidence of homozygous calls at the subsequent steps of SNP identification. Sequencing procedure yielded the average of nine million sequencing paired-end reads for each rapeseed line. After the read filtering procedures, the average of 7.5 million paired-end reads per line were mapped to the rapeseed genome. The average depth of sequenced fragments was of 37.3 ± 8.7. Based on these data, we identified 160,257 biallelic SNPs using the GATK SNP caller. From these SNPs, we removed the low covered SNPs (DP < 10), rare variants (MAF < 0.03), as well as the positions containing more than 30% of missing data. Since rapeseed is an amphidiploid plant, we further discarded SNPs with a high heterozygosity rate (>0.4), as previously described [37]. As a result, 12,226 SNPs remained for further analyses.

**Figure 1:**
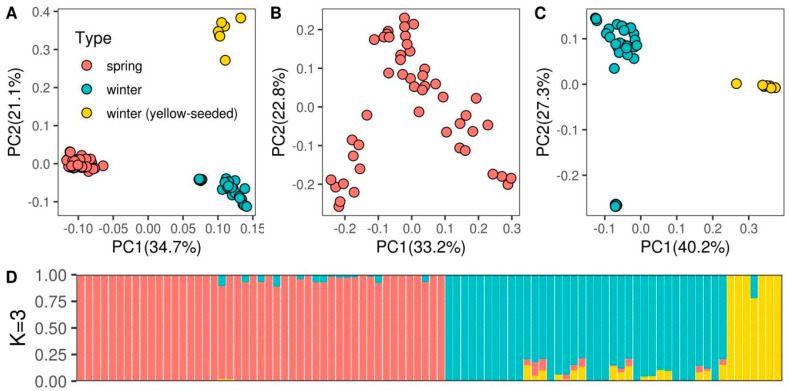
Population structure of Russian rapeseed lines. Population structure assessed using principal component analysis for the whole cohort (**A**), spring (**B**), and winter (**C**) types separately. Red dots correspond to spring rapeseed accessions. Blue dots correspond to winter rapeseed accessions. Yellow dots correspond to yellow-seeded winter rapeseed accessions. (**D**) Population clustering of rapeseed lines based on the admixture component of each accession, the bar colors correspond to the dot colors in panel A.

Visualization of the general genetic variation in 90 Russian rapeseed lines using the principal component analysis (PCA) based on 12,226 genome-wide SNPs revealed three main clusters (Figure 1A). The first principal component, explaining 34.7% of the total genetic variance, separated the winter and spring rapeseed lines. The second principal component, explaining 21.1% of the total genetic variance, split the winter rapeseed in accordance with seed phenotype data. The separate analysis of the winter ecotypes revealed additional population structure, for the spring ecotype no clear clusters were identified (Figure 1B,C).

We further assessed the number of potential subpopulation clusters among the 90 rapeseed lines using the ADMIXTURE software package [35]. The significant drop of cross-validation error was observed when the studied cohort was split into two, three, five, and seven clusters (Figure S1A and Figure 1D). Splitting 90 lines into two clusters matched the winter and spring rapeseed ecotypes. At cluster number K = 3 the winter rapeseed plants split into two subpopulations, yellow-seeded and dark-seeded, while all spring rapeseed lines clustered together (Figure 1D and Figure S1B). Another significant drop of cross-validation error corresponded to cluster numbers K = 5 and K = 7. The corresponding clusters, however, did not separate in accordance with the phenotype data (Figure S1B).

**Figure 2:**
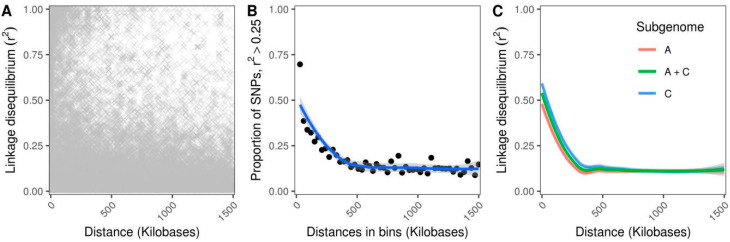
Linkage disequilibrium (LD) decay in the studied cohort of rapeseed lines. (**A**) LD decay across potential genetic marker (SNP) pairs. Each cross corresponds to the *r^2^* value between a pair of SNPs. (**B**) Proportion of SNP pairs with *r^2^* > 0.25 was calculated for the whole genome. Each dot indicates the proportion of SNP pairs in the 30 kb bin. (**C**) LD decay for A and C subgenomes. Colored lines on panels B and C represent loess curves. Grey markers correspond to the 95% confidence interval.

We determined the relationship between the linkage disequilibrium (LD) decay and the distance between SNPs in the genomes of 90 lines directly based on the squared correlation coefficient (*r^2^*) and on the proportion of SNP pairs with the *r^2^* value greater than 0.25 within 30 kb bins (Figure 2A,B). We further calculated the LD decay separately for the rapeseed A and C subgenomes corresponding to turnip (*Brassica rapa*) and cabbage (*Brassica oleracea*), respectively, and the entire (A + C) rapeseed genome (Figure 2C). Overall, the estimated length of LD blocks at *r^2^* = 0.25 threshold was 218.8 ± 14.5 kilobases (kb) for the entire rapeseed genome, 177.1 ± 19.5 kb for the A subgenome, and 265.1 ± 13.4 kb for the C subgenome (Figure 2C).

**Figure 3:**
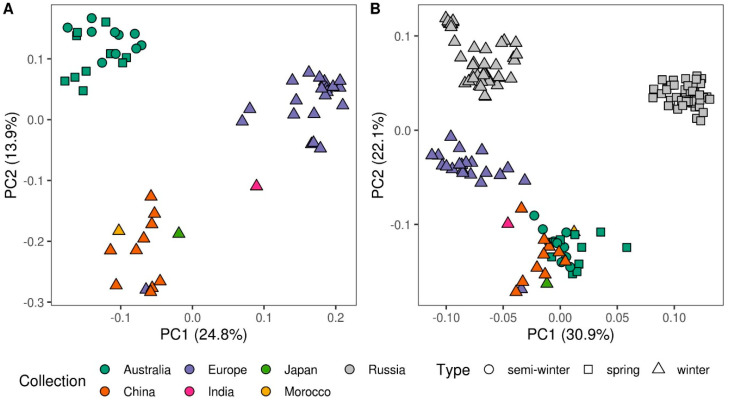
Principal component analysis (PCA) plots reflecting the population structure of Russian and foreign rapeseed collections. PCA analysis was performed using 20,848 SNPs polymorphic in at least one of the datasets (Russian and foreign rapeseed datasets). (**A**) The population structure of foreign lines used in this study. (**B**) The comparison of the population structure of Russian and foreign rapeseed lines. Colors correspond to the collection, the shape indicates the rapeseed ecotype.

To compare the genetic diversity of rapeseed lines from Russian collection with the rapeseed accessions from worldwide collection, we used the whole-genome sequencing data collected by Malmberg et al. [37] for 54 geographically and ecotypically diverse rapeseed lines. We reanalyzed these data following the same procedure as was used for the Russian rapeseed collection resulting in 4,037,572 unfiltered SNPs. Next, these SNPs were joined with the unfiltered 160,257 SNPs from the present study with subsequent filtering of the two datasets using sequencing depth and missing value filters. As a result, we identified 20,848 SNPs polymorphic in at least one of the two datasets. Visualization of the genetic diversity of the 54 foreign lines using PCA revealed three distinct clusters (Figure 3A). One cluster corresponded to the Australian semi-winter and spring rapeseeds. The other two clusters contained winter rapeseed lines: One geographically diverse, containing lines grown in China, Japan, Morocco, India, and one European line, and the other containing only European winter rapeseed lines. In its turn, the combined analysis of rapeseed lines from Russian and foreign collections revealed clear genetic separation of Russian rapeseed accessions, especially the ones corresponding to the spring ecotype (Figure 3B). This separation remained stable when either 20,848 SNPs polymorphic in at least one of the datasets, Russian or foreign (Figure 3B), or 5749 SNPs polymorphic in both datasets were used (Figure S2).

### 3.2. Glucosinolate Phenotyping and Genetic Association Analysis

**Figure 4:**
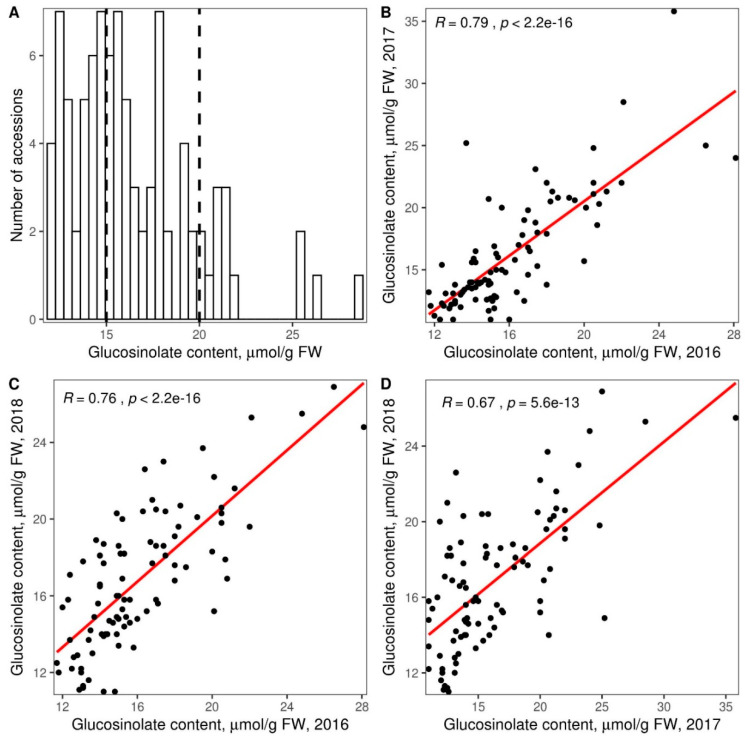
Glucosinolate content distribution in the studied rapeseed cohort. (**A**) Histogram depicting average glucosinolate content values for three years. Dashed lines divide low, middle, and high glucosinolate lines according to the All-Russian Research Institute of Oil Crops (VNIIMK) classification. (**B**–**D**) Correlation of glucosinolate content for three vegetational seasons. Each dot corresponds to a plant sample. Regression lines are shown in red.

To assess what genetic variants are linked to the quantitative variation of glucosinolate level in the Russian rapeseed collection, we measured the total glucosinolate content using palladium colorimetric analysis. The measurements were carried out for all 90 rapeseed lines from Russian collection during the three-year period to account for ecological variability (Table S1, Figure S3). The glucosinolate content correlated positively and significantly between years with the Pearson’s correlation coefficient ranging from 0.67 to 0.79 (Pearson correlation, *p* < 0.0001; Figure 4A–C). The glucosinolate content in the lines ranged from 11.0 to 35.8 micromoles per gram of fresh weight with the average value of 16.3 across three years. This concentration range spans all three rapeseed glucosinolate content levels according to the VNIIMK classification: Low (<15 micromoles per gram), middle (between 15 and 25 micromoles per gram), and high (>25 micromoles per gram) (Figure 4A).

**Figure 5:**
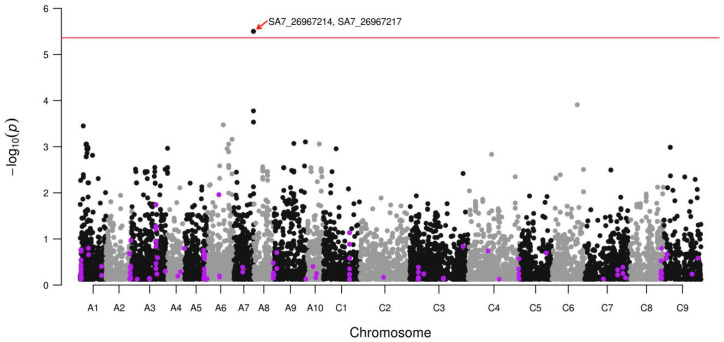
Manhattan plot showing SNP markers associated with glucosinolate content. Each dot corresponds to a single SNP. Red line corresponds to the Bonferroni adjusted significance threshold. Purple dots correspond to SNPs demonstrated to be significantly associated with glucosinolate content in the previous studies.

We next searched for the genetic determinants of the glucosinolate content variation in rapeseed lines from the VNIIMK collection using the mixed linear model approach. The model included five principal components and kinship matrix to account for the population structure and kin relationships, respectively. We applied the model to the glucosinolate measurements for each year (Figure S4), calculating the cumulative significance of genetic associations using the Irwin–Hall distribution. The analysis yielded two neighboring SNPs (SA7_26967214 and SA7_26967217) located on the chromosome A7 significantly associated with glucosinolate levels after the Bonferroni correction (Figure 5). The significance level for the next five SNPs ranged according to the observed association significance that passed the soft significance threshold of 0.0005 (Table S3). Notably, our analysis revealed no significant association signals for the SNPs linked to glucosinolate concentration in the foreign rapeseed lines in the previous studies [24,25,26,42,43] (Figure 5). The total phenotypic variation of glucosinolate levels explained by the two most significant SNPs varied from 13.8 to 20.4% across three years (Table S3). The five SNPs passing the soft significance threshold from 8.5 to 23.4% explained the glucosinolate variation (Table S3).

According to rapeseed genome annotation, the two SNPs significantly associated with glucosinolate concentrations in the Russian rapeseed lines are localized within the intergenic region between the genes encoding the transcription termination factor MTERF2 chloroplastic-like and the U-box domain-containing protein 35-like (Table S4). Furthermore, the analysis of the genes located within the window of 100 kb upstream and downstream of the two SNPs significantly associated with glucosinolate content revealed the genes encoding the histone acetyltransferase HAC1 and BES1/BZR1 homolog protein 4-like (Table S5), respectively, linked to glucosinolate content in the previous studies [45,46]. Among the five marginally significant SNPs, one was annotated as a missense variant (SA1_4407039) and one as a synonymous variant (SA6_21541176) within the genes encoding the uncharacterized protein BNAA01G06520D and derlin-2.1 protein, respectively (Table S4). The remaining three SNPs localized outside the gene regions, with one (SA1_4407039) being located 31.8 kb downstream of another gene encoding the γ-glutamyl peptidase 1-like protein (Table S5), previously connected to the control of glucosinolate concentration [47,48].

## 4. Discussion

We applied the genotyping-by-sequencing (GBS) approach to the description of the population structure of the Russian collection of rapeseed from the Pustovoit All-Russian Research Institute of Oil Crops (VNIIMK) and to the search for the potential genetic markers (SNPs) associated with an agronomically important trait, namely, glucosinolate content. Most of the studies related to high-throughput genotyping were performed using DNA microarrays [6,25,42] but the GBS approach has also been utilized in rapeseed in order to find associations for agronomically important traits [14] and to describe population structure and genetic diversity [9,37]. Despite the fact that GBS and microarray-based genotyping usually detect the same QTLs [15,17], the GBS approach in some cases allows detecting rare or previously unknown genetic variants compared to microarrays due to the standard design of the latter [16,17]. In addition, the use of the GBS technique allows to reduce genotyping costs [15] and to avoid the ascertainment bias in the diversity analysis studies based on relatively non-representative plant cohorts [17]. In the present study, the GBS approach indeed helped to better assess the genetic diversity of rapeseed lines from the VNIIMK collection, given its genetic difference from the international rapeseed varieties used in the microarray design.

We used the ZS11 genome to map sequencing reads since it is one of the most recent and fully assembled and annotated *B. napus* genomes [4]. Using BWA aligner in the combination with the GATK tools yielded 160,257 SNPs for the studied cohort, of which 12,226 high-quality positions passed the quality filters. This number of SNPs is approximately 8–7 times lower than that obtained using a similar approach in the previous studies [9,14], which could result from the relatively stringent filters applied. It should be noted that in addition to the filters related to SNP quality and the percentage of missing data, we removed all sites that demonstrated high heterozygosity rates (>40%). The observed high amount of heterozygous positions is the consequence of the amphidiploid nature of the rapeseed genome which results in the presence of highly homologous regions often leading to the read misalignment [37]. Thus, the heterozygosity filter should be applied for sequencing-derived SNPs [10].

Population structure analysis (Figure S1 and Figure 1) demonstrated that the two clusters revealed by the ADMIXTURE algorithm divided the population into spring and winter rapeseed ecotypes. Such separation was quite expected since spring and winter rapeseeds represent the ecotypes with different growth regimes. Seeds of spring plants are sown in spring in contrast to the seeds of winter plants that are sown in early autumn and require vernalization [8]. Growth regime (ecotype) was previously demonstrated to be the major factor explaining the genetic structure in rapeseed [8,11,12,49,50] and remains such for the studied cohort of Russian rapeseed lines (Figure 1A). This is explained by significant breeding efforts made to adapt rapeseed to environmental conditions which in turn limited the gene pool and resulted in separation of spring and winter ecotypes during rapeseed breeding history [8,37,42]. Notably, when we split the collection into three clusters, one of the clusters distinguished a small subpopulation of yellow-seed winter rapeseed. The similar separation resulted from the principal component analysis, namely by the second PC (Figure 1A). It is quite a new observation that the genetic data would yield the clear separation of yellow and dark-seeded rapeseeds. However, since the color of the seeds is generally explained by a few genomic regions, such separation could also be a result of a recent breeding history of yellow-seeded accessions analyzed in the present study. Unfortunately, additional clustering of the studied cohort to five and seven sub-populations did not show any clear correspondence with the phenotype or biochemical data. Probably such segregation could be explained by the common relatively recent ancestry of these lines.

Our estimate of LD decay within chromosomes and subgenomes conventionally based on the *r^2^* parameter revealed a subtle difference between the two subgenomes, one derived from *B. rapa* (A subgenome) and one from *B. oleracea* (C subgenome), with the high level of homology [4]. Namely, the LD decay was shorter for A and longer for C subgenome, at the linkage cutoff *r^2^* = 0.25 (Figure 2C). The observed difference in the LD pattern between the subgenomes was consistent with the previous studies conducted using non-Russian rapeseed collections [6,11,51]. This difference could further be related to the fact that the chromosomes of C subgenomes are longer on average compared to the A subgenome [4].

Since our study is the first to describe the genetic variability in a substantial number of rapeseed lines of Russian selection, we compared the genetic diversity of the lines from Russian collection with 54 diverse lines from around the world [37] (Figure 3A). While non-Russian lines clustered according to their geographic origins and ecotype in agreement with the previous analyses [8,11,12,49], all of them differed drastically in their genetic composition from all 90 rapeseed accessions from the Russian collection. Which in turn indicates a great potential of this collection for breeders who may use it in order to increase the diversity of collections and mine for new variations of traits. Furthermore, such difference between Russian and international collections could be used further for a deeper investigation of the rapeseed breeding history. The differences in the genetic composition between the rapeseed accessions of Russian selection and those representing world’s diversity imply differences in the genetic determinants of the important traits. This, in turn, provides an opportunity to study the genetic mechanisms underlying these traits from a different angle. To test this idea, we conducted the genome-wide analysis of associations between the genetic variants found in 90 Russian rapeseed accessions and the quantitative variation in their glucosinolate content measured during three years of cultivation. Using the mixed linear model approach, we identified two SNP markers significantly associated with the glucosinolate content, as well as five additional SNP markers using a more relaxed threshold of *p* < 0.0005 (Table S3, Figure 5). These markers explained from 8.5 to 23.4% of phenotypic variance, which is consistent with the previous works where genetic markers explained from 4–8 and up to 35–42% of glucosinolate content variation [24,25,42,43]. The small number of associated SNPs identified could be a result of using a quite limited number of 90 accessions. Another possible consequence of studying 90 lines for association mapping could probably be related to the fact that the markers identified did not overlap with the regions identified in the previous studies. Furthermore, previously identified loci did not show any trend to display significant *p*-values at both thresholds. Additionally, the difference between the current and the published studies could potentially be attributed to two factors. First, all lines used in our study had a relatively low glucosinolate content varying from 11 to 36 micromoles per gram of fresh weight with the average of 16 (Figure 4). By contrast, in the published studies, the seed glucosinolate content varied from 8–28 and up to 131–146 micromoles per gram of fresh weight [24,42,43] making these samples more diverse in terms of glucosinolate content. Second, the observed difference in the genetic markers of the glucosinolate content between Russian and non-Russian rapeseed collections could reflect the difference in the genetic composition between them revealed in our study. Thus, the addition of lines with higher glucosinolate content from other collections on one hand could increase the number and significance of the loci associated with glucosinolate content. On the other hand, the loci that were found in the present study would be probably masked by the previously identified loci strongly associated with glucosinolate content.

To find new candidate genes that potentially contribute to the control of glucosinolate content, we studied the annotation of genes located 100 kb upstream and downstream of the significant SNPs. Such threshold was selected since, first, it was previously used in the similar studies in rapeseed [7,52,53]. Second, more than 30% of SNP pairs located within the 100 kb frame demonstrated the *r^2^* value of more than 0.25 (Figure 2B). Previously, this approach yielded several candidate genes located in the vicinity of the glucosinolate-associated SNPs. The first group of the candidate genes included the ones encoding enzymes directly responsible for glucosinolate metabolism, namely, AOP3 enzymes involved in the final steps of glucosinolate biosynthesis [26], glucosinolate side chain biosynthetic enzymes MAM1 and MAM3 [43,54], and glucosinolate transporter GTR2 [24,25]. The second group includes genes encoding proteins involved in the regulation of glucosinolate content, namely, the HAG1 transcription factor controlling aliphatic glucosinolate biosynthesis [26,42] and MYB28 and MYB34 family transcription regulators. In our study, none of these genes localized close to the identified markers. Nonetheless, our analysis revealed several genes located close to the markers identified in our study that could be related to the pathways controlling glucosinolate content (Table S5). Specifically, two significant markers, SA7_26967214 and SA7_26967217, were located 38.1 and 75.3 kb downstream of the genes encoding the histone acetyltransferase HAC1 and BES1/BZR1 homolog protein 4-like, respectively (Table S5). The HAC1 encoding histone acetyltransferase was shown to be involved in leaf senescence in plants [46]. Notably, the HAC1 *Arabidopsis thaliana* knockout mutants demonstrated the repression of genes involved in the metabolism of glucosinolate [46]. The BZR family proteins include transcription factors involved in the brassinosteroid signaling pathway. It was demonstrated that BZR1 and BES1 of the BZR family regulate glucosinolate biosynthesis through brassinosteroid-dependent signaling [45]. Thus, the data on the candidate genes obtained in the present study could potentially support the role of these genes in the regulation of glucosinolate content in rapeseed plants. Another SNP marker SA1_4407039 was located 31.8 kb downstream of the gene encoding the γ-glutamyl peptidase 1-like protein (Table S5). Previously, γ-glutamyl peptidases (GGP1 and GGP3) were shown to be involved in the metabolism of glucosinolates [47,48], with the *ggp1* and *ggp3* knockout mutants demonstrating impaired glucosinolate profiles compared to the wild-type *A. thaliana* plants [48].

## 5. Conclusions

In conclusion, this study is one of the first with the focus on high-throughput genotyping of a Russian rapeseed collection. The obtained data made possible the description of the population structure of the rapeseed collection from VNIIMK, the leading institute in rapeseed breeding in Russia and allowed comparing the genetic composition of the Russian collection with that of the international lines elucidating substantial genetic differences between the Russian and international rapeseed collections. Notably, the obtained genotype data as well as the information on VNIIMK collection could facilitate future studies focused on the investigation of diversity and breeding history of rapeseed. Furthermore, the provided data could promote breeders to use rapeseed accessions from VNIIMK collection in the breeding programs in order to enrich genetic diversity of their collections. The collection of the three-year phenotype data for the important agronomic trait, glucosinolate content, allowed us to find potential genetic markers and identify new candidate genes related to this trait. Importantly, genetic markers linked to the glucosinolate content in the Russian collection did not overlap with the ones identified using international rapeseed lines. Several candidate genes identified in the present study were previously discussed in terms of glucosinolate biosynthesis that in turn additionally supports their role in this process and speak in favor of significance of SNPs identified. Furthermore, genes that were not previously shown to be associated with glucosinolate could serve as a potential candidate for a deeper investigation of glucosinolate biosynthesis and its genetic control in the future studies. This finding highlights the high value of regional collections in describing the full spectrum of genetic variants linked to a phenotypic trait. Since the SNPs identified explain a substantial amount of glucosinolate variance, they could be applied for development of the marker assisted selection programs aimed at reducing the glucosinolate content in the rapeseed. Thus, the data and results obtained in the present study will, therefore, facilitate the understanding of rapeseed genetic diversity and the genetic control of glucosinolate content, finally leading to the development of improved marker-associated breeding programs in Russia and worldwide.

## Supplementary Materials

The following are available online at https://www.mdpi.com/2073-4425/11/8/926/s1. Figure S1: Population structure assessment using the admixture software. (A) Estimated cross-validation error value for possible clusters from 1 to 10. Drop of the cross-validation error indicates the optimal number of possible subpopulations. (B) Admixture bar plots reflecting the subpopulations at K = 3, 5, 7, each bar corresponds to the rapeseed line, colors indicate the proportion of subpopulation admixtures in each individual. At K = 3, red bars correspond to spring rapeseed accessions, blue bars correspond to winter rapeseed accessions, and yellow bars correspond to yellow-seeded winter rapeseed accessions; Figure S2: PCA plots depicting the population structure of Russian and foreign rapeseed collections. PCA analysis was performed using 5749 SNPs polymorphic in both datasets (Russian and foreign rapeseed datasets). (A) The population structure of foreign lines used in this study. (B) The comparison of the population structure of Russian and foreign rapeseed. Colors correspond to the collection of origin, shape indicates rapeseed ecotype; Figure S3: The distribution of mean glucosinolate content across three years for the rapeseed accessions under study. Dashed lines indicate low, middle, and high glucosinolate content groups; Figure S4: Manhattan plots and corresponding quantile-quantile plots for the estimated −log10 (*p*-value) for each of the three years as well as the mean value of glucosinolate concentration. Red arrows indicate SNPs SA7_26967214, SA7_26967217; Table S1: Ecotype, origin, collection and glucosinolate concentration of the rapeseed lines used in the present study; Table S2: List of the sequenced rapeseed lines with corresponding replicate names and barcodes; Table S3: List of SNPs, significantly associated with the glucosinolate content; Table S4: Annotation of SNPs located not more than 5 kb away from genes and within genes; Table S5: Annotation of SNPs located not more than 100 kb away from the candidate genes.
