# Supplementary figures and images for: Genetic Characterization of Russian Rapeseed Collection and Association Mapping of Novel Loci Affecting Glucosinolate Content
Source: Genes (Basel). 2020 Aug 12;11(8):926. doi: 10.3390/genes11080926 (PMC7465703; doi:10.3390/genes11080926)

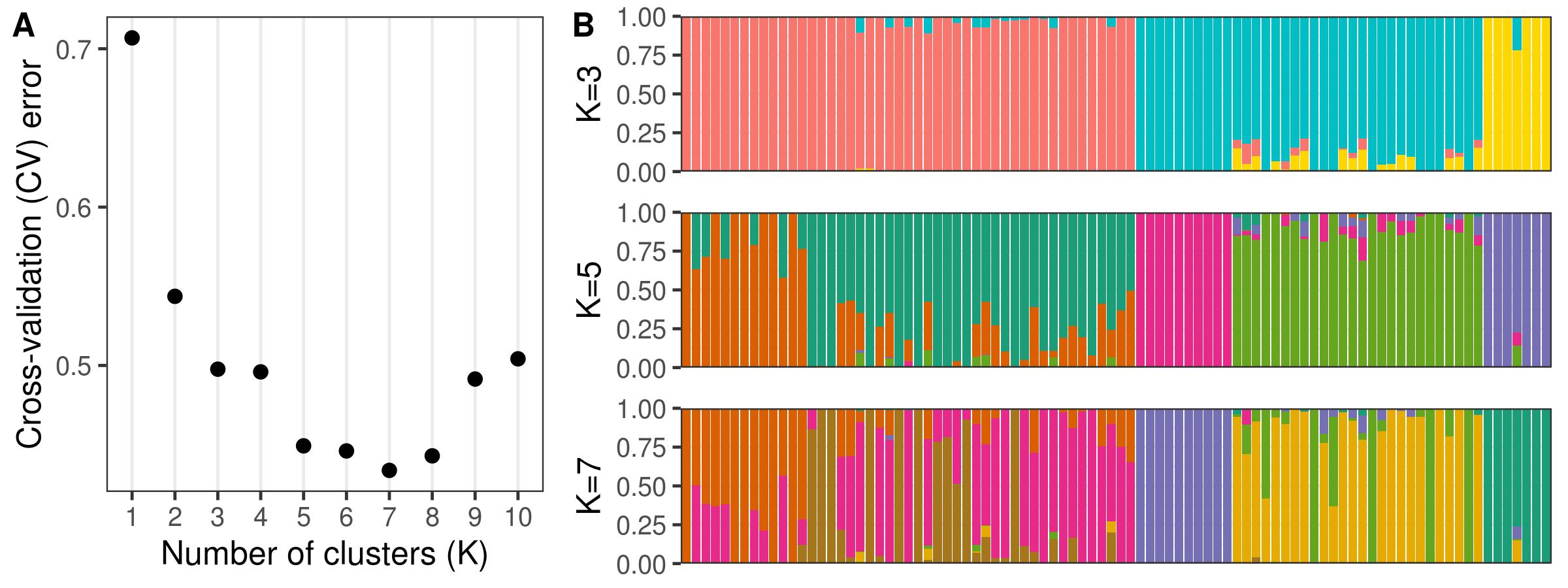

Supplement: Supplementary file 1 [file genes-11-00926-s001.zip › genes-879589-supplementary materials/FigureS1.jpeg]

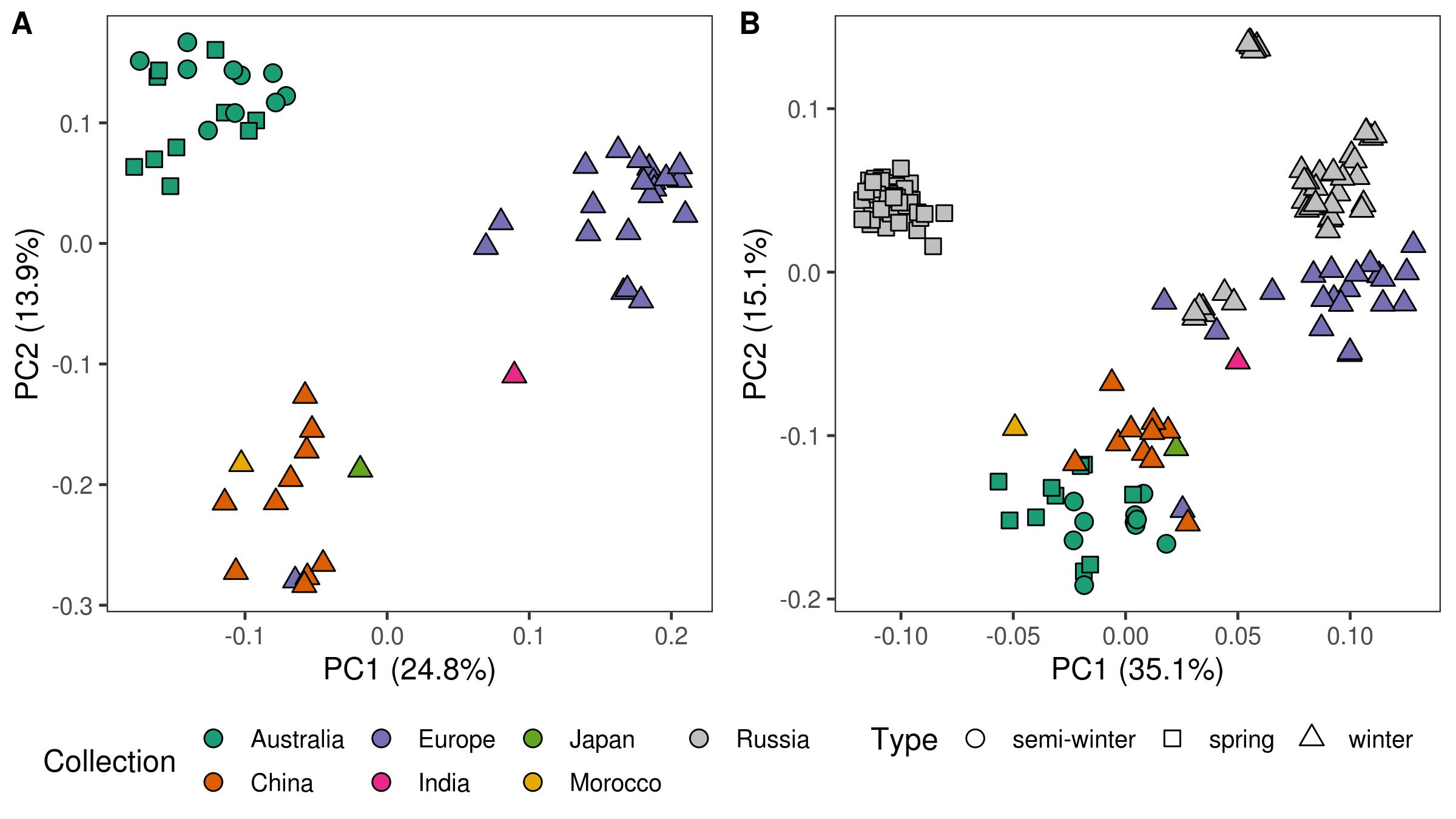

Supplement: Supplementary file 1 [file genes-11-00926-s001.zip › genes-879589-supplementary materials/FigureS2.jpeg]

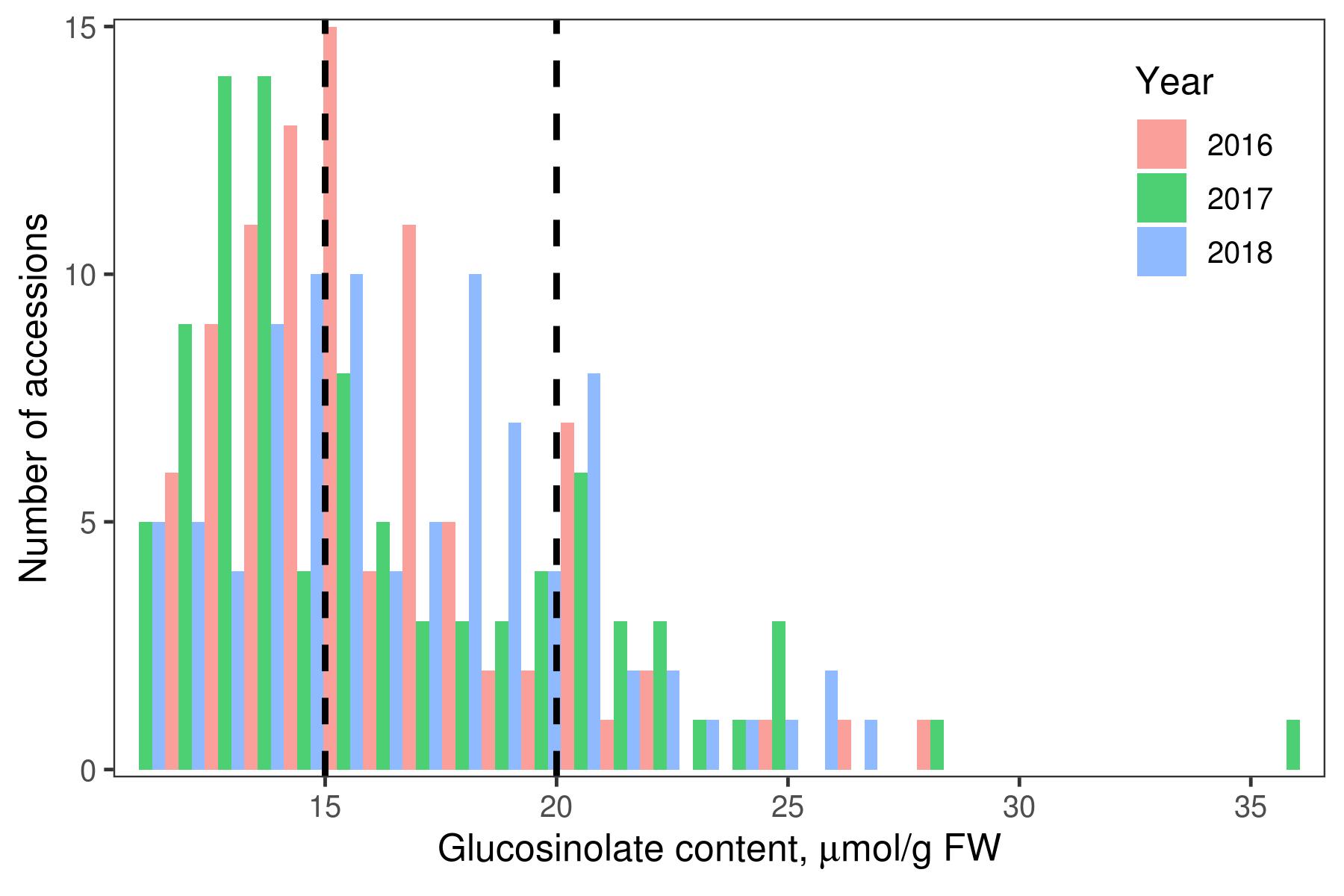

Supplement: Supplementary file 1 [file genes-11-00926-s001.zip › genes-879589-supplementary materials/FigureS3.jpeg]

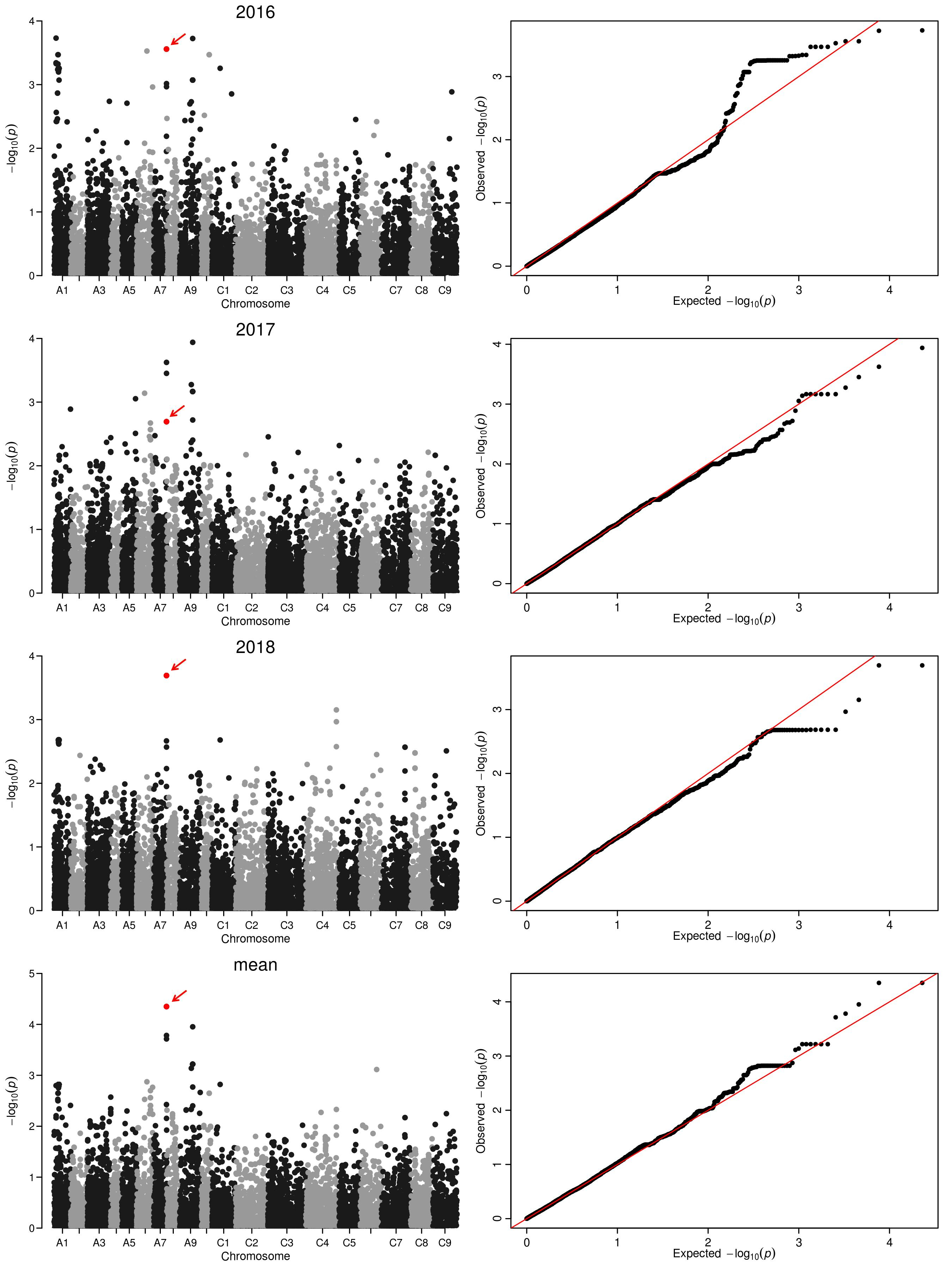

Supplement: Supplementary file 1 [file genes-11-00926-s001.zip › genes-879589-supplementary materials/FigureS4.jpeg]
